# A Perilous Plunge: A Unique Case of Rectal Foreign Body

**DOI:** 10.7759/cureus.78904

**Published:** 2025-02-12

**Authors:** Shehzadi Rimsha, Danish Aslam, Subas Ali, Shehanshah Muhammed Arqam, Ayesha Kausar

**Affiliations:** 1 General Surgery, Sindh Government Hospital New Karachi, Karachi, PAK; 2 General Surgery, Civil Hospital Karachi, Karachi, PAK

**Keywords:** dangerous sexual practices, general surgery and colorectal surgery, perianal surgery, risky sexual behaviors, unusual rectal foreign objects

## Abstract

Rectal foreign bodies (RFBs) are foreign bodies in the rectum that pose a challenge to the surgeons in the emergency department. We report a case of an RFB; the patient was a 30-year-old man who put a glass bottle into his rectum on his own. The 10-cm bottle was successfully retrieved through the transanal approach without complication. The patient had no sign of injury or bleeding after the procedure. The patient was stable and was discharged with counseling to avoid such conduct. An effective approach for extraction is to have a structured protocol encompassing prompt diagnosis, extraction techniques, and post-removal assessment. In addition, there is a psychological aspect to RFBs that requires counseling and patient education.

## Introduction

Rectal foreign bodies (RFBs) are an emergency clinical problem that challenges surgeons in the emergency department. Data on the actual epidemiology of RFB is unavailable as the condition remains underreported; however, the incidence seems to be increasing in the present years [1]. The literature shows its prevalence among middle-aged males aged 30 to 40 years, irrespective of their sexual orientation [2]. The reasons for inserting an RFB are sexual stimulation, accidental insertion, or, rarely, assault [3].

The patient with RFB may be asymptomatic and have severe abdominal pain, bleeding, or signs of peritonitis, depending on the type of object and the time it had been retained [4]. Other complications are fatal and include perforation and sepsis. If RFB is diagnosed early and managed properly, morbidity and mortality can be prevented [3].

Management strategies for RFBs are influenced by factors including the object's size, shape, material, and location within the rectum or colon. The treatment is urgent extraction of the foreign body since the retention time of the object increases the risk of injury to the wall and bowel perforation [4,5]. The patient's local and systemic conditions, the foreign body's characteristics, and the available resources must be considered for extraction. Different techniques are available, where transanal extraction stands out, which can be performed in an operating room with regional or general anesthesia, as long as the patient's condition and the characteristics of the object allow it. Another noninvasive method is flexible sigmoidoscopy. When these methods fail, transanal minimally invasive surgery, a minimally invasive alternative, is used [6]. Exploratory laparotomy is indicated in cases where there is migration of the object, suspicion of colorectal perforation, associated abscesses, or transanal extraction has failed [7].

## Case presentation

A 30-year-old unmarried male, employed as a laborer and with no known comorbidities, presented to the emergency department with complaints of a foreign body inserted through the anus. The patient denied any history of assault and admitted to multiple prior episodes of similar insertions. On examination, the patient was vitally stable. Abdominal examination revealed a soft, non-tender abdomen with a palpable foreign body around the umbilical region. Digital rectal examination showed normal anal tone with a glass bottle palpable in the rectum from the anal verge. The laboratory investigations revealed an elevated total leukocyte count of 13.9 x 10^9/L, which may indicate inflammation (Table 1).

**Table 1:** Laboratory investigation results Anti-HCV: anti-hepatitis C virus, HBsAg: hepatitis B surface antigen, INR: international normalized ratio

Cells	Patient results	Unit	Normal range
Hemoglobin	15.2	gram/dl	13-18
Total leukocyte count	13.9	x 10^3/L	4.5-11.0
Platelets	301	x 10^3/L	150-300
Blood urea nitrogen	14	mg/dL	6-20
Creatinine	0.8	mg/dL	0.5-0.9
INR	1.21	-	<1.5
Anti-HCV	Non-reactive	-	-
HBsAg	Non-reactive	-	-

**Figure 1:**
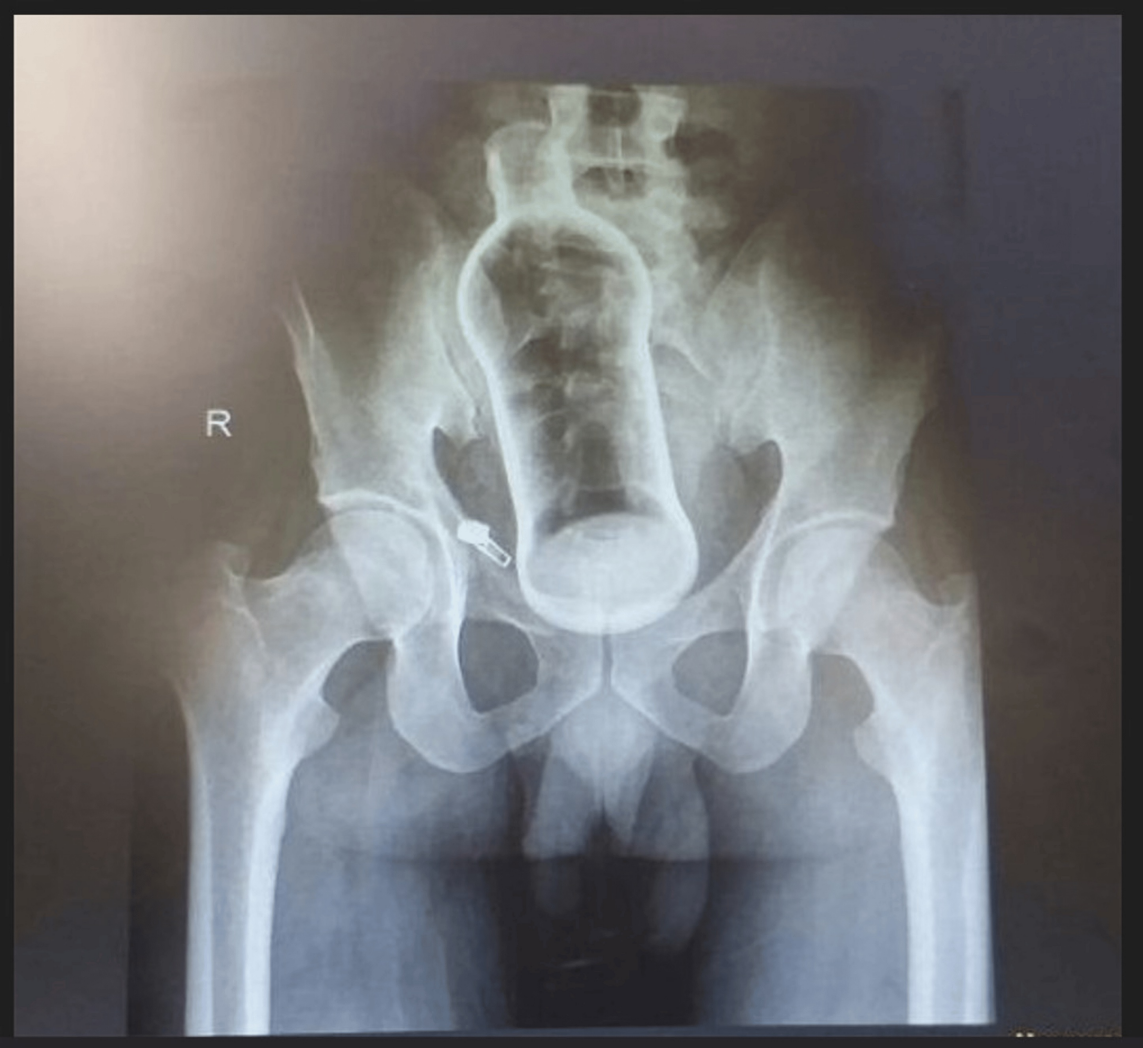
Pelvic X-ray anteroposterior view revealing a foreign body

The abdominal ultrasound did not show any free fluid but excessive gases. Chest and abdominal plain radiographs confirmed the presence of a glass bottle measuring approximately 10 cm within the rectum (Figure 1).

**Figure 2:**
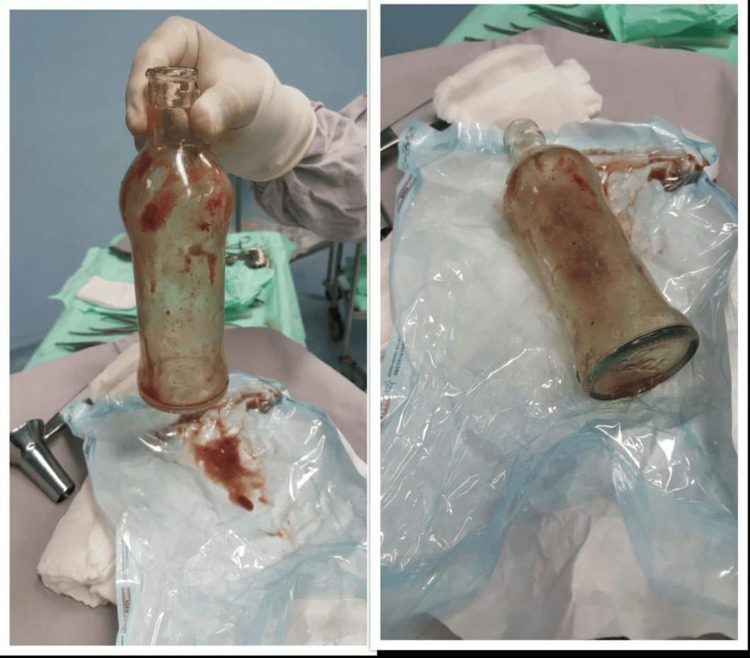
Glass bottle extracted

The patient was then prepared for examination under anesthesia in the operating room. The glass bottle was removed by putting gentle abdominal pressure in a lithotomy position (Figure 2). This technique is referred to as transanal extraction. A follow-up proctoscopy examination showed no mucosal lesions or hemorrhages. The patient had an uneventful postoperative recovery and was counseled to refrain from such practices.

## Discussion

RFB insertion is both a diagnostic and therapeutic challenge. RFB cases are relatively rare in the emergency department, but their number has risen in recent years [2]. It could also be due to changing social perceptions about sexual orientation and acceptance of people who live different lifestyles. Most cases reported have been associated with sexual pleasure, accounting for 78% to 100% of cases [8]. However, patients often delay seeking medical help due to shame or embarrassment, which can increase the risk of complications [9]. In this case, the patient’s history of several similar episodes implies that recurrence is likely, so appropriate preventive strategies are required.

Diagnostic imaging, such as radiographs and ultrasonography, is very important in localizing and determining the size and type of foreign bodies and often guides the management [3]. Radiography helped determine the size and presence of a glass bottle in our patient. The principal objective of managing RFBs is removing the object without causing trauma to the rectal mucosa. Transanal extraction is the first choice among non-surgical options [6]. Large spherical-shaped objects can be treated with obstetric vacuum devices [10]. In our case, abdominal pressure was applied under general anesthesia, and the glass bottle was successfully extracted without complications.

After extraction, the rectal mucosa should be evaluated for any possible injuries and injury to the sphincter. While some authors recommend postoperative endoscopic examination such as sigmoidoscopy, some believe that clinical examination may suffice when the patient is asymptomatic and shows no signs of perforation [4,8]. In the present case, no mucosal damage was observed in proctoscopy, and the patient remained asymptomatic at follow-up.

The patient had a history of RFB insertion, which signifies the behavioral and psychological factors in such cases. A psychiatric assessment and subsequent counseling are recommended to avoid the repetition of such episodes in the future [11]. However, patients tend to avoid a visit to a psychologist due to the stigma attached to mental health problems. Therefore, healthcare professionals should be sensitive to this issue and provide information on the adverse health effects that result from such behavior.

The management of cases of RFBs should include ethical considerations. This allows people to seek medical attention on time [12]. Preventions to such incidents can be awareness campaigns that help the public know the dangers of inserting objects in the rectum.

## Conclusions

The extraction of RFBs is a complex procedure. The case illustrated the difficulties associated with managing RFBs in a patient with multiple past episodes of similar insertions. An effective approach is a structured protocol encompassing prompt diagnosis, extraction techniques, and post-removal assessment. Counseling and patient education should also address psychological factors to avoid a recurrence. Preventive measures are very important, including educational activities to make people aware of the risks that are involved with inserting foreign bodies in the rectum. Such implementations will, therefore, enable optimization of patients’ outcomes and prevent future repetition of such incidents.
